# Is ultrasound-guided hip aspiration more successful than fluoroscopic-guided aspiration in diagnosing prosthetic joint infection?

**DOI:** 10.5194/jbji-8-151-2023

**Published:** 2023-05-09

**Authors:** Emily A. Treu, Daniel M. Cushman, John C. Wheelwright, Brenna E. Blackburn, Masaru Teramoto, Michael J. Archibeck

**Affiliations:** 1 Department of Orthopedic Surgery, University of Utah, 590 Wakara Way, Salt Lake City, UT 84108, USA; 2 Department of Physical Medicine & Rehabilitation, University of Utah, 590 Wakara Way, Salt Lake City, UT 84108, USA

## Abstract

**Introduction**: aspiration of total hip arthroplasty (THA) is commonly performed to assist in the diagnosis of prosthetic joint infection (PJI). This study aimed to determine whether fluoroscopic- or ultrasound- guided hip aspiration differs in the ability to acquire synovial fluid and in the accuracy of diagnosing infection. **Methods:** all THA aspirations performed between 2014 and 2021 at our institution were retrospectively identified. Aspirations were classified as successful or dry. If successful, the volume of fluid obtained was recorded. The sensitivity and specificity of hip aspiration in identifying PJI were calculated with four methods: (1) culture results excluding saline lavage, (2) culture results including saline lavage, (3) 2018 Musculoskeletal Infection Society (MSIS) International Consensus Meeting (ICM) criteria, and (4) 2021 European Bone and Joint Infection Society (EBJIS) criteria. Analyses were performed using Student's t test or Wilcoxon rank sum for continuous variables and chi-squared or Fisher's exact test for categorical variables. **Results:** 290 aspirations were included (155 fluoroscopic-guided and 135 ultrasound-guided). Success of aspiration ( >0.5 mL) was more common in the ultrasound cohort (69 %) than fluoroscopy (53 %) ( p<0.0055 ). When successful, more volume was obtained in the ultrasound cohort (mean 13.1 mL vs. 10.0 mL; p=0.0002 ). Ultrasound-guided aspiration was more sensitive than fluoroscopy in diagnosing PJI using culture results excluding saline lavage (85 % vs. 73 %; p=0.03 ), culture results including saline lavage (85 % vs. 69 %; p=0.001 ), 2018 MSIS-ICM criteria (77 % vs. 52 %; p=0.02 ), and 2021 EBJIS criteria (87 % vs. 65 %; p=0.02 ). Ultrasound-guided aspiration was more specific than fluoroscopy in diagnosing PJI using 2021 EBJIS criteria (100 % vs. 96 %; p=0.001 ). **Conclusions:** ultrasound-guided aspiration is more frequently successful and yields more fluid than fluoroscopic-guided aspiration of THA. Ultrasound-guided aspiration is more sensitive in diagnosing PJI than fluoroscopy using culture data, 2018 MSIS-ICM criteria, and 2021 EBJIS criteria.

## 1 Introduction

Prosthetic joint infection (PJI) is a major cause of morbidity and mortality following total hip arthroplasty (THA) (Berend et al., 2013; Shahi et al., 2017; Zmistowski et al., 2013). Infection rates in primary THA are approximately 1 % and higher in revision surgery (Berbari et al., 1998; Kurtz et al., 2008; Pulido et al., 2008). Accurate diagnosis of PJI is important to guide appropriate treatment but can be difficult due to lack of definitive testing (Carli et al., 2019; Fernández-Sampedro et al., 2017). The Musculoskeletal Infection Society (MSIS) issued diagnostic criteria in 2011 (Parvizi et al., 2011) and an updated version in 2018 (Parvizi et al., 2018). These criteria have previously been considered the gold standard for diagnosing PJI (97.7 % sensitivity, 99.5 % specificity) (Parvizi et al., 2018). In 2021, the European Bone and Joint Infection Society (EBJIS) issued new diagnostic criteria, supported by MSIS, which many consider to be the new gold standard for diagnosing PJI due to a higher sensitivity and easier clinical decision-making (Mcnally et al., 2021; Sigmund et al., 2022; Sousa et al., 2023).

Aspiration is critical in making the diagnosis of PJI. Aspiration cultures can identify causative organisms, and synovial fluid cell counts contribute to the diagnosis of PJI through MSIS minor criteria. Total hip aspiration is typically performed under image guidance. Fluoroscopic-guided hip aspiration is the most prevalent modality (Ali et al., 2006; Barrack and Harris, 1993; Cheung et al., 1997; Battaglia et al., 2011; Cross et al., 2014; Fehring and Cohen, 1996; Glithero et al., 1993; Gould et al., 1990; Itasaka et al., 2001; Johnson et al., 1988; Kanthawang et al., 2021; Kraemer et al., 1993; Lachiewicz et al., 1996; Levitsky et al., 1991; Lieberman et al., 1993; Mulcahy et al., 1996; Phillips and Kattapuram, 1983; Pons et al., 1999; Randelli et al., 2018; Roberts et al., 1992; Somme et al., 2003; Spangehl et al., 1999; Taylor and Beggs, 1995; Tigges et al., 1993; Williams et al., 2004), however, ultrasound has gained popularity in recent years (Battaglia et al., 2011; Eisler et al., 2001; Randelli et al., 2018; Van Holsbeeck et al., 1994). Ultrasound guidance allows for more precise needle placement and better visualization of soft tissue structures, extraarticular fluid collections, and intraarticular effusions (Long et al., 2012; Parvizi et al., 2018). Ultrasound has an improved safety profile due to the absence of radiation exposure or contrast agents and is more cost-effective (Randelli et al., 2018).

This work is a subanalysis of a larger group excluding native hips and specifically investigating the diagnosis of PJI in arthroplasty patients (Roesly et al., 2022). The objectives of our study were to (1) identify whether fluoroscopic or ultrasound guidance is more successful in obtaining fluid when aspirating a total hip arthroplasty, and (2) identify the sensitivity and specificity of each technique in diagnosing PJI.

## 2 Methods

### 2.1 Data collection

We performed a retrospective chart review of all aspirations of total hip replacements completed at a single academic institution from May 2014 to February 2021, using the Current Procedural Terminology (CPT) codes 20610 and 20611. Aspirations of total hip arthroplasties were included if they were performed under image guidance for suspicion of PJI or desire to rule out PJI prior to revision surgery. Patients with more than one aspiration were included in the study as separate data points. Aspirations were also excluded if they were incorrectly coded or aborted due to patient intolerance.

Chart review was performed to verify diagnosis and collect relevant information. Demographic information was documented including age, gender, and body mass index (BMI). All available data points used to calculate a 2018 MSIS International Consensus Meeting (ICM) and 2021 EBJIS score were collected (Parvizi et al., 2018; Mcnally et al., 2021). This included the preoperative finding of a draining sinus and preoperative inflammatory markers. Aspirations were recorded as a success ( ≥0.5 mL) or dry aspiration ( <0.5 mL). The cutoff of 0.5 mL of fluid was to exclude aspirations where fluid was likely a result of needle trauma rather than intraarticular fluid, and in this case the fluid was not sent for analysis. For aspirations where 0.5 to 1 mL of synovial fluid was obtained, there was often only enough fluid to test for cultures but not cell counts. We recorded the volume of fluid obtained and aspiration results available including white blood cell (WBC) count, polymorphonuclear (PMN) percentage, synovial alpha-defensin (if available), gram stain, and cultures. We documented intraoperative culture results, presence of intraoperative purulence, and histology findings of the subsequent revision surgery if performed following the aspiration attempt, and we included these findings in the postoperative 2018 MSIS-ICM and 2021 EBJIS score calculations.

### 2.2 Aspiration technique

All aspirations were performed at an outpatient orthopedic clinic by physicians with fellowship training in musculoskeletal radiology, sports medicine, or pain medicine. The use of fluoroscopy or ultrasound guidance for the aspiration was based on provider preference and availability. Local anesthetic was administered into subcutaneous tissue, avoiding intraarticular administration to prevent any bacteriostatic effects of lidocaine. An 18-, 20-, or 22- gauge needle was used to perform the aspiration based on provider discretion. The amount of fluid aspirated in the syringe was recorded by the physician. In general, the goal of each aspiration was to aspirate as much fluid from the joint as possible. Percutaneous biopsies were not performed.

Fluoroscopic-guided aspirations were performed using an anterolateral approach in the supine position. The needle was advanced under image guidance, aiming toward the head–neck junction of the prosthesis until the needle could be felt contacting metal. If fluid was obtained upon entry into the joint, then no contrast material was injected. If no fluid was obtained initially, then intraarticular position of the needle was confirmed with injection of air or iodinated contrast agent. If blood was aspirated, the needle was repositioned. In eight cases, fluid lavage using a saline flush was performed due to insufficient quantity on initial aspiration.

Ultrasound-guided aspirations began with an initial inspection for the presence of a periarticular fluid collection or joint effusion. Most cases were performed through an anterior approach in the supine position. However, a posterior approach in the prone position was performed in select patients where preliminary ultrasound suggested higher fluid yield posteriorly. If no fluid was visualized, aspiration was performed via an anterior approach. Multiple attempts to obtain fluid using needle repositioning were performed if initial attempts were unsuccessful. Fluid lavage was not used in any cases of ultrasound-guided aspiration. Additionally, no needle guide was used to perform the aspirations.

### 2.3 Aspiration analysis

We compared the average needle size used to perform the aspiration between fluoroscopy and ultrasound. We compared percentage of dry aspiration attempts between cohorts. We compared the average amount of fluid obtained with each modality, first including dry aspiration attempts and second excluding them. In this analysis, aspirations that required saline lavage (eight), were recorded as dry aspirations due to the inability to obtain fluid initially.

### 2.4 Sensitivity/specificity analysis

Four different analyses were performed to determine the sensitivity and specificity of each aspiration imaging modality in diagnosing PJI: (1) culture results excluding saline lavage, (2) culture results including saline lavage, (3) 2018 MSIS-ICM criteria, and (4) 2021 EBJIS criteria. Sensitivity [true positive/(true positive + false negative)] and specificity [true negative/(true negative + false positive)] were determined based on results of a “screening test” compared to results of a “gold standard test.”

In the first analysis (culture results excluding saline lavage), we included all patients who had both a preoperative aspiration and resulted intraoperative cultures. Patients were excluded who had dry aspiration attempts, saline lavage performed, no aspiration cultures sent, no revision surgery performed, or no cultures sent at the time of revision surgery. A positive screening test result was defined as an aspiration culture with organism growth. A positive gold standard test result was defined as two or more intraoperative cultures with organism growth. In the case of only one positive intraoperative culture, the aspiration was considered a true positive if it grew the same organism as the intraoperative culture, it was considered a false positive if it grew a different organism than the intraoperative culture, or it was considered a true negative if it offered no growth in the setting of only a single positive intraoperative culture.

In the second analysis (culture results including saline lavage), we included all patients who had a preoperative aspiration culture, including those that underwent saline lavage, and resulting intraoperative cultures. Previous literature suggests that culture results after saline lavage are reliable (Li et al., 2019; Partridge et al., 2018). Patients were excluded who had dry aspiration attempts, no aspiration cultures sent, no revision surgery performed, or no cultures sent at the time of revision surgery. The definitions for a positive screening test and gold standard test were the same as the first analysis.

**Table 1:** 2018 MSIS-ICM criteria described by Parvizi et al. (2018).

Major criteria (at least one of the following)				Decision
Two positive cultures of the same organism				Infected
Sinus tract with evidence of communication to the joint or visualization of the prosthesis				Infected
Minor criteria			Score	Decision
Preoperative diagnosis	Serum	Elevated CRP or D-dimer	2	
		Elevated ESR	1	≥6 infected
	Synovial	Elevated synovial WBC or LE	3	2–5 possibly infected^a^
		Positive alpha-defensin	3	0–1 not Infected
		Elevated synovial PMN %	2	
		Elevated synovial CRP	1	
Intraoperative diagnosis^a^		Positive histology^c^	3	≥6 infected
		Positive purulence	3	4–5 inconclusive^b^
		Single positive culture	2	≤3 not infected

2018 Musculoskeletal Infection Society (MSIS) definition of prosthetic joint infection. CRP – C-reactive protein, ESR – erythrocyte sedimentation rate, LE – leukocyte esterase, PMN – polymorphonuclear, and WBC – white blood cell. ^a^ For patients with inconclusive minor criteria, operative criteria can also be used to fulfill the definition for PJI. ^b^ Consider further molecular diagnostics such as next-generation sequencing. ^c^ Histology was defined as positive if there were more than five neutrophils per high-power field in five high-power fields ( ×400 ).

In the third analysis (2018 MSIS-ICM criteria), we included all patients being worked up for chronic PJI ( >12 weeks from the index procedure). Patients being worked up for acute PJI ( <12 weeks) were excluded due to differing cell count and lab value thresholds for acute vs. chronic infection based on these criteria. According to the 2018 MSIS-ICM criteria, PJI is diagnosed if one major criterion is present or a score of ≥6 points using minor criteria is calculated. Major criteria include two positive cultures of the same organism and presence of a sinus tract with evidence of communication to the joint or visualization of the prosthesis. Minor criteria are determined based on preoperative serum and synovial fluid analysis and intraoperative findings (Parvizi et al., 2018) (Table 1). We used the cutoff values for chronic PJI as described by Parvizi et al. (2018) (Table 2). Using the 2018 MSIS-ICM criteria, a positive screening test result was defined as a positive aspiration culture or one that met MSIS minor criteria preoperatively (score ≥6 ). A positive gold standard test result was defined as one that fulfilled MSIS-ICM major or minor criteria (score ≥6 ), taking intraoperative data into account as well.

**Table 2:** Cutoff values for specific PJI markers based on 2018 MSIS-ICM criteria [11].

Markers	Chronic ( >90 d)	Acute ( <90 d)
Serum CRP (mg dL^-1^)	1.0	10
Serum D-dimer (ng mL^-1^)	860	860
Serum ESR (mm h^-1^)	30	–
Synovial WBC count (cells µ L^-1^)	3000	10 000
Synovial PMN (%)	80	90
Synovial CRP (mg L^-1^)	6.9	6.9
Synovial alpha-defensin (signal-to-cutoff ratio)	1.0	1.0

**Table 3:** 2021 EBJIS Criteria described by McNally et al. (2021).

	Infection unlikely (all findings negative)	Infection likely (two positive findings)	Infection confirmed (any positive finding)
Clinical and blood workup			
Clinical features	Clear alternative reason for implant dysfunction (e.g., fracture, implant breakage, malposition, tumor).	Radiological signs of loosening within the first 5 years after implantation. Previous wound healing problems. History of recent fever or bacteraemnia. Purulence around the prosthesis.	Sinus tract with evidence of communication to the joint or visualization of the prosthesis.
C-reactive protein		>10 mg L^-1^ (1 mg dL^-1^)	
Synovial fluid cytological analysis			
Leukocyte count(cells µ L^-1^)	≤1500	>1500	>3000
PMN (%)	≤65%	>65%	>80%
Microbiology			
Aspiration fluid		Positive culture	
Intraoperative (fluid and tissue)	All cultures negative	Single positive culture	≥2 positive samples with the same microorganism
Sonication (CFU ml)	No growth	>1 CFU mL^-1^ of any organism	>50 CFU mL^-1^ of any organism
Histology			
High-power filed ( 400× magnification)	Negative	Presence of ≥5 neutrophils in a single HPF	Presence of ≥5 neutrophils in ≥5 HPF
			Presence of visible microorganisms
Others			
Nuclear imaging	Negative three-phase isotope bone scan	Positive WBC scintigraphy	

In the fourth analysis (2021 EBJIS criteria), we included all patients being worked up for acute or chronic PJI, as these criteria do not distinguish thresholds by acuity of infection. The 2021 EBJIS criteria allocate diagnostic tests to three groups: infection confirmed, infection likely, and infection unlikely. PJI is confirmed if any of the below criteria are present: sinus tract with evidence of communication to the joint or visualization of the prosthesis, leukocyte count >3000 cells µ L^-1^, PMN >80% , positive alpha-defensin immunoassay or lateral-flow assay, ≥ two intraoperative positive samples with the same microorganism, >50 colony forming units (CFU) mL^-1^ of any intraoperative organism, presence of ≥ five neutrophils in ≥ five high-power field, or presence of visible microorganisms. PJI is likely if two diagnostic criteria are met within this category and unlikely if all findings are negative (Table 3). Using the 2021 EBJIS criteria, a positive screening test result was defined as a positive aspiration culture or one that met EBJIS confirmed criteria preoperatively. A positive gold standard test result was defined as one that fulfilled EBJIS confirmed criteria, taking intraoperative data into account as well.

### 2.5 Statistical analysis

Patient characteristics were summarized descriptively and compared between cohorts. Continuous variables were summarized as mean (SD) and range and compared using Student's t tests or Wilcoxon rank sum tests, depending on the distribution. Categorical variables were summarized as N (%) and compared using chi-squared or Fisher's exact tests. The equality of distributions was compared between cohorts using Kolmogrov–Smirnoff test. Statistical significance was defined as p<0.05 for all tests. All statistical analysis was completed using SAS 9.4 (Cary, NC).

**Figure 1:**
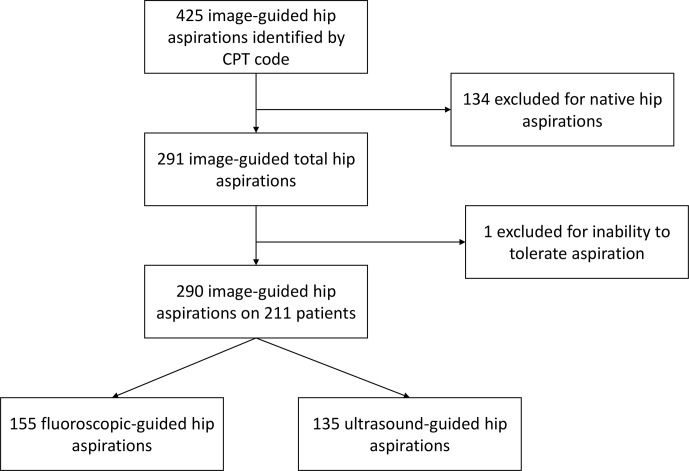
Flowchart of aspirations included in this study.

## 3 Results

Of image-guided hip aspirations, 425 were identified by CPT code; 134 of these were native hip aspirations and thus excluded from our study. One total hip aspiration was excluded as it was aborted due to the patient's inability to tolerate the aspiration. This left 290 image-guided total hip aspirations in 211 patients available for review. Of these, 155 total hip aspirations were performed with fluoroscopic guidance and 135 with ultrasound guidance (Fig. 1).

### 3.1 Patient demographics

The average age of patients in our cohort was 62.4 (range of 23–92), with 55 % female and 45 % male. No difference in age ( p=0.277 ), sex ( p=0.470 ), or BMI ( p=0.096 ) was found between the fluoroscopic- and ultrasound- guided groups (Table 4).

**Table 4:** Patient demographics compared between cohorts.

	Fluoroscopy	Ultrasound	
	( N=155 )	( N=135 )	
	mean (SD)	mean (SD)	P value
Age	63.2 (13.3)	61.5 (13.6)	0.277
BMI	31.9 (8.5)	30.3 (7.3)	0.096
	N (%)	N (%)	P value
Sex			
Female	83 (53.6)	78 (57.8)	0.470
Male	72 (46.5)	57 (42.2)	

**Figure 2:**
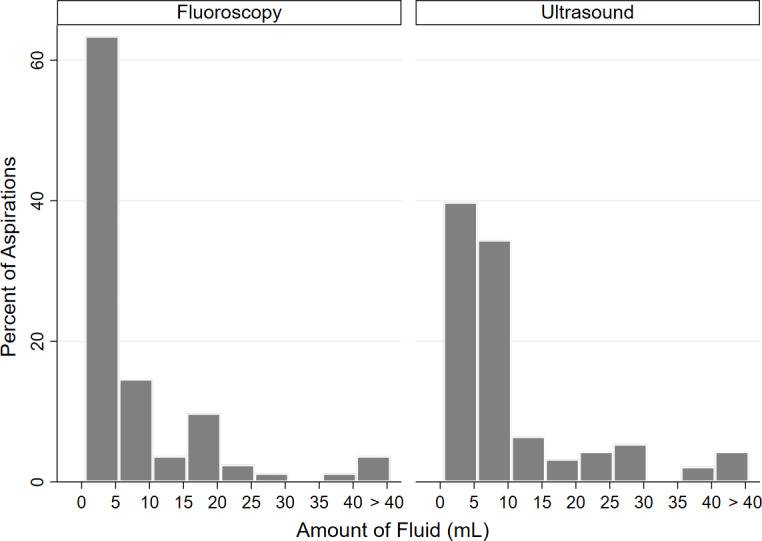
Histogram of fluid distributions of each cohort.

### 3.2 Aspiration characteristics 

An 18-gauge needle was used in 78.1 % of fluoroscopic-guided aspirations and 72.4 % of ultrasound-guided aspirations ( p=0.2636 ). Fluoroscopic guidance resulted in a dry aspiration 47.1 % of the time, while ultrasound resulted in a dry aspiration 31.1 % of the time ( p<0.0055 ). An average of 5.2 mL was obtained with fluoroscopic guidance, and an average of 9.0 mL of fluid was obtained with ultrasound guidance ( p=0.0001 ). When dry aspirations were excluded, an average of 10.0 mL was obtained with fluoroscopic guidance, and an average of 13.1 mL of fluid was obtained with ultrasound guidance ( p=0.0002 ) (Table 5).  Of those aspirations where fluid was obtained, the distribution of fluid volume was significantly different between cohorts ( p=0.005 ). Of fluoroscopic-guided aspirations, 63.5 % yielded <5 mL of fluid, 14.5 % yielded 5–10 mL, and 22 % yielded >10 mL. Of ultrasound-guided aspirations, 39.8 % yielded <5 mL of fluid, 34.4 % yielded 5–10 mL, and 25.8 % yielded >10 mL (Fig. 2).

**Table 5:** Aspiration characteristics compared between cohorts.

	Fluoroscopy ( N=155 )	Ultrasound ( N=135 )	
	N (%)	N (%)	P value
Needle size			
18	121 (78.1)	97 (72.4)	0.2636
20/22	34 (21.9)	37 (27.6)	
Dry taps	73 (47.1)	42 (31.1)	0.0055
	Mean (SD, median, IQR)	Mean (SD, median, IQR)	P value
Fluid amount (mL)	5.3 (15.2, 1.0, 3.0)	9.0 (18.1, 3.0, 10.0)	0.0001
Fluid amount excluding dry taps (mL)	10.0 (19.7, 3.0, 7.0)	13.1 (20.6, 8.0, 9.0)	0.0002

### 3.3 Sensitivity/specificity analysis

In the first analysis using culture results excluding saline lavage, 47 aspirations in each cohort met the inclusion criteria. The sensitivity of the fluoroscopy cohort in the identification of PJI was 73 % and the ultrasound cohort was 85 % ( p=0.02 ). No difference was seen in specificity between cohorts (94 % vs. 93 %; p=0.77 ) (Table 6). In the second analysis using culture results including saline lavage, 51 fluoroscopic aspirations and 47 ultrasound aspirations met the inclusion criteria. The sensitivity of the fluoroscopy cohort in the identification of PJI was 69 % and the ultrasound cohort was 85 % ( p=0.001 ). No difference was seen in specificity between cohorts (91 % vs. 93 %; p=0.78 ) (Table 6). In the third analysis using 2018 MSIS-ICM criteria, 152 fluoroscopic and 134 ultrasound-guided aspirations met inclusion criteria. The sensitivity of the fluoroscopy cohort was 52 % and the ultrasound cohort was 77 % ( p=0.02 ). No difference was seen in specificity between cohorts (98 % vs. 99 %; p=0.66 ) (Table 6). In the fourth analysis using 2021 EBJIS criteria, 155 fluoroscopic and 135 ultrasound-guided aspiration aspirations met inclusion criteria. The sensitivity of the fluoroscopy cohort was 65 % and the ultrasound cohort was 87 % ( p=0.02 ). The specificity of the fluoroscopy cohort was 96 % and the ultrasound cohort was 100 % ( p=0.001 ) (Table 6).

**Table 6:** Sensitivity and specificity analysis (95 % confidence intervals).

	Fluoroscopy	Ultrasound	P value
Using culture results only			
	N=47	N=47	
Sensitivity	73 % (51, 96)	85 % (69, 100)	0.02
Specificity	94 % (85, 100)	93 % (83, 100)	0.77
Using culture results with saline lavage			
	N=51	N=47	
Sensitivity	69 % (46, 91)	85 % (69, 100)	0.001
Specificity	91 % (82, 100)	93 % (83, 100)	0.78
Using 2018 MSIS-ICM criteria			
	N=152	N=134	
Sensitivity	52 % (37, 68)	77 % (63, 92)	0.02
Specificity	98 % (96, 100)	99 % (97, 100)	0.66
Using EBJIS criteria			
	N=155	N=135	
Sensitivity	65 % (51, 78)	87 % (77, 97)	0.02
Specificity	96 % (83, 100)	100 % (100, 100)	0.001

### 3.4 Culture data

We evaluated the concordance of aspiration cultures and intraoperative cultures within each cohort (Tables 7 and 8).

The fluoroscopy cohort had 10 positive aspiration cultures with at least two positive intraoperative cultures (true positive). One aspiration culture was positive for coagulase-negative *Staphylococcus* (CoNS) with one positive intraoperative culture growing the same organism (true positive). One aspiration culture was positive for *Serratia* in the setting of negative intraoperative cultures (false positive). One aspiration culture was positive for CoNS, while the intraoperative culture grew *Cutibacterium acnes* in only one culture (false positive). Four aspiration cultures yielded no growth, while at least two intraoperative cultures were positive for the same organism (false negative). Twenty-two aspirations yielded negative cultures with negative intraoperative cultures (true negative). Eight aspirations yielded negative cultures with only one positive intraoperative culture (true negative). Of these, the most common intraoperative bacteria to grow was coagulase-negative * Staphylococcus* (Table 7).

**Table 7:** Culture data of the fluoroscopic-guided aspiration cohort.

	Aspirate culture	Intraop culture (no. positive culture/total cultures)
True positive	*S. aureus*	*S. aureus *(3/3)
	*S. aureus*	*S. aureus *(2/3)
	*S. aureus*	*S. aureus *(2/5), CoNS (1/5)
	CoNS	CoNS (3/3)
	CoNS	CoNS (3/4)
	CoNS	CoNS (1/1)
	CoNS	CoNS (4/6), *S. aureus *(1/6)
	*Escherichia coli*	*E. coli *(4/4)
	*Enterococcus faecalis*	*E. faecalis *(4/4)
	*C. glabrata*	*S. aureus *(4/6), *C. glabrata *(2/6), diphtheroid (1/6)
	*C. acnes; *CoNS	*C. acnes (5/5)*
False positive	*Serratia*	No growth
	CoNS	*C. acnes* (1/3)
False negative	No growth	Anaerobes (2/3)
		Anaerobes (3/5)
		*E. faecalis * (2/3), *E. coli *(1/3)
		*E. faecium * (2/3), *C. albicans *(1/3)
True negative	No growth (30)	No growth (22)
		CoNS (1/3), *S. aureus* (1/3), *C. acnes* (1/3)
		*C. acnes * (1/3)
		CoNS (1/4)
		CoNS (1/3)
		CoNS (1/5)
		CoNS (1/6)
		CoNS (1/3)
		CoNS (1/3)

^*^ One row per patient. CoNS – coagulase-negative *Staphylococcus*.

**Table 8:** Culture data of the ultrasound-guided aspiration cohort.

	Aspirate culture	Intraop culture (no. positive culture/total cultures)
True positive	*S. aureus*	*S. aureus *(4/5)
	*S. aureus*	*S. aureus *(3/3)
	*S. aureus*	*S. aureus *(1/2), CoNS (1/2), * C. acnes *(1/2)
	*S. aureus*	*S. aureus *(2/3)
	CoNS	CoNS (2/3)
	CoNS	CoNS (1/1)
	CoNS	CoNS (3/4)
	CoNS	CoNS (2/5),* C. acnes *(1/5)
	CoNS	CoNS (5/5),* Kocuria* (1/5)
	CoNS	CoNS (5/6)
	CoNS	CoNS (1/4)
	*Klebsiella*	*Klebsiella *(2/3)
	*E. faecium*	*E. faecium *(3/3),* Kocuria *(1/3)
	*Corynebacterium*	*Corynebacterium *(1/5)
	*Pseudomonas*	*Pseudomonas *(1/4),* C. acnes *(1/4)
	*C. acnes*	*C. acnes *(4/4)
	*Eubacterium*	*Eubacterium *(2/3)
False positive	*C. avidum* *S. aureus*	No growth CoNS (1/3)
False negative	No growth	CoNS (2/5),* C. acnes *(1/5)
		*Corynebacterium *(3/3), *C. acnes *(3/4)
True negative	No growth (25)	No growth (22)
		*C. acnes *(1/3), *S. aureus *(1/3)
		*C. acnes *(1/2)
		*C. acnes *(1/5)

Note: one row per patient. CoNS – coagulase-negative *Staphylococcus*.

The ultrasound cohort had 12 positive aspiration cultures with at least two positive intraoperative cultures (true positive). Five aspirations had positive culture growth with one intraoperative culture growing the same organism (true positive). One aspiration culture was positive for *Cutibacterium avidum* in the setting of negative intraoperative cultures (false positive). One aspiration culture was positive for *Staphylococcus aureus*, while the intraoperative culture grew coagulase-negative * Staphylococcus * in only one culture (false positive). Three aspiration cultures yielded no growth, while at least two intraoperative cultures were positive for the same organism (false negative). Twenty-two aspirations yielded negative cultures with negative intraoperative cultures (true negative). Three aspirations yielded negative cultures with only one positive intraoperative culture (true negative). Of these, the most common intraoperative bacteria to grow was *C. acnes* (Table 8).

We then evaluated the dry aspirations in each cohort which had subsequent intraoperative culture growth (Table 9). Twenty of the 73 dry aspirations in the fluoroscopy group (27.4 %) had positive intraoperative cultures. Of these, four grew bacteria on only one culture, while the remaining 16 had two or more positive cultures. Six of the 42 dry aspirations in the ultrasound group (14.3 %) had positive intraoperative cultures, all with two or more positive cultures which grew the same organism.

Lastly, we evaluated the number of intraoperative cultures taken when an aspiration was positive or negative for culture growth. Those aspirations with no subsequent revision surgery were excluded from this analysis. Of the aspirations with no culture growth, 24.1 % had no subsequent intraoperative cultures, 7.6 % had one intraoperative culture, 7.0 % had two intraoperative cultures, and 61.4 % had three or more intraoperative cultures sent for analysis. Of the aspirations with positive culture growth, 0 % had no subsequent intraoperative cultures sent, 6.1 % had one intraoperative culture, 0 % had two intraoperative cultures, and 93.9 % had three or more intraoperative cultures sent for analysis ( p=0.0004 ).

## 4 Discussion

Our study is the largest patient series comparing fluoroscopic- and ultrasound-guided total hip aspiration. We found that ultrasound resulted in significantly fewer dry aspirations than fluoroscopy (31.1 % vs. 47.1 %; p=0.0055 ). Successful aspiration and ability to analyze synovial fluid are critical components in making the diagnosis of PJI, utilizing both culture results and calculation of a MSIS-ICM or EBJIS score. Dry aspiration increases the possibility of obtaining a false negative result (Kanthawang et al., 2021; Schulz et al., 2021). Christensen et al. (2022) compared sensitivity and specificity of cultures between dry aspirations ( <0.5 mL) requiring saline lavage and successful aspirations. They found that the dry aspiration cohort had a significantly higher rate of negative aspiration cultures followed by positive intraoperative cultures. Our study had a total of 26 patients (20 fluoroscopy, six ultrasound) with dry aspiration attempts followed by positive intraoperative cultures. While many surgeons consider a dry aspiration attempt to be reassuring, we recommend exercising caution and scrutiny in this patient population given our findings. We believe that ultrasound-guided aspiration reduces the frequency of uncertainty in PJI diagnosis compared to fluoroscopy due to its higher success rate in obtaining fluid. If fluoroscopic aspiration is first performed and results in a dry aspiration attempt, we recommend performing a repeat ultrasound-guided aspiration.

**Table 9:** Positive intraoperative culture results for dry aspirations in each cohort.

Fluoroscopy (no. positive culture/total cultures)	Ultrasound (no. positive culture/total cultures)
*C. acnes *(1/5)	CoNS (2/2)
*C. acnes* (1/5)	CoNS (4/5)
*C. acnes *(1/3),* S. mitis *(1/3)	*C. acnes *(2/3)
*C. acnes *(3/4)	*Corynebacterium *(4/4)
*C. acnes *(4/5)	*Corynebacterium *(2/2), *S. aureus *(1/2)
*C. acnes *(1/3),* Pseudomonas *(1/3)	*Finegoldia *(2/2),* S. aureus *(1/2)
*S. aureus *(4/4)	
*S. aureus *(1/6)	
CoNS (1/3)	
CoNS (3/3)	
*C. albicans *(2/3)	
*C. albicans *(2/3)	
*Klebsiella *(2/3),* C. albicans *(1/3)	
*Pseudomonas *(1/3),* C. acnes *(1/3)	
*Eubacterium *(2/3)	
*Corynebacterium *(4/4)	
*S. lugdunensis *(3/4)	
*Enterococcus *(2/3)*, C. albicans *(1/3)	
Anaerobes (2/2)	
Group B* Streptococcus *(2/3)	

Note: one row per patient. CoNS – coagulase-negative *Staphylococcus*.

**Table 10:** Number of intraoperative cultures taken separated by aspiration culture growth.

Aspiration culture growth			
	Negative	Positive	
	N (%)	N (%)	P value
0 cultures	38 (24.1)	0 (0)	0.0004
1 culture	12 (7.6)	2 (6.1)	
2 cultures	11 (7.0)	0 (0)	
3+ cultures	97 (61.4)	31 (93.9)	

Ultrasound-guided aspiration was not only more successful but also yielded significantly more fluid than fluoroscopy (9.0 mL vs. 5.2 mL; p=0.0001 ), even when excluding dry aspiration attempts (13.1 mL vs. 10.0 mL; p=0.0002 ). The ability of ultrasound to directly visualize and target fluid collections in or around the hip likely explains the greater mean volume of fluid obtained when compared to fluoroscopy. Rockov et al. (2020) showed that, in the diagnosis of PJI, aspiration cultures were more likely to correlate with intraoperative cultures at higher aspiration volumes. The higher volume of fluid obtained with ultrasound could therefore contribute to our finding that ultrasound was more sensitive than fluoroscopy in diagnosing PJI.

Many studies have evaluated the accuracy of fluoroscopic-guided hip aspiration in identifying PJI, with sensitivities ranging from 12 %–100 % and specificities of 75 %–100 % (Ali et al., 2006; Barrack and Harris, 1993; Cheung et al., 1997; Battaglia et al., 2011; Cross et al., 2014; Fehring and Cohen, 1996; Glithero et al., 1993; Gould et al., 1990; Itasaka et al., 2001; Johnson et al., 1988; Kanthawang et al., 2021; Kraemer et al., 1993; Lachiewicz et al., 1996; Levitsky et al., 1991; Lieberman et al., 1993; Mulcahy et al., 1996; Phillips and Kattapuram, 1983; Pons et al., 1999; Randelli et al., 2018; Roberts et al., 1992; Somme et al., 2003; Spangehl et al., 1999; Taylor and Beggs, 1995; Tigges et al., 1993; Williams et al., 2004). Fewer studies have reported on the accuracy of ultrasound in identifying PJI, with sensitivities ranging from 0 %–100 % and specificities of 74 %–96 % (Battaglia et al., 2011; Eisler et al., 2001; Randelli et al., 2018; Van Holsbeeck et al., 1994). Comparative studies of the two imaging techniques in the setting of THA are limited. Only two known studies directly compared results of fluoroscopy and ultrasound. Battaglia et al. (2011) reported a 69 % sensitivity and 94 % specificity of ultrasound compared to a 27 % sensitivity and 75 % specificity of fluoroscopy in a cohort of 60 total hip aspirations. Randelli et al. (2018) reported an 89 % sensitivity and 94 % specificity of ultrasound compared to a 60 % sensitivity and 81 % specificity of fluoroscopy in a cohort of 52 total hip aspirations (Randelli et al., 2018). While most studies use intraoperative cultures as the gold standard for diagnosing PJI, two prior studies used MSIS-ICM criteria as the gold standard in diagnosing PJI of THA (Kanthawang et al., 2021; Randelli et al., 2018). Kanthawang et al. (2021) evaluated fluoroscopic-guided aspiration of 202 total hips using 2018 MSIS-ICM criteria. They reported a 64 % sensitivity and 78.5 % accuracy using aspiration cultures and 74.2 % sensitivity and 82.1 % accuracy using synovial polymorphonuclear neutrophil (PMN) % (Kanthawang et al., 2021). Randelli et al. (2018) used 2013 MSIS-ICM criteria as the gold standard, and these results are stated above. To our knowledge, no prior studies have used EBJIS criteria as the gold standard method to compare fluoroscopic- and ultrasound-guided total hip aspiration. In our study, we found that ultrasound-guided aspiration was more sensitive than fluoroscopy in diagnosing PJI using culture results excluding saline lavage (85 % vs. 73 %; p=0.03 ), culture results including saline lavage (85 % vs. 69 %; p=0.001 ), 2018 MSIS-ICM criteria (77 % vs. 52 %; p=0.02 ), and 2021 EBJIS criteria (87 % vs. 65 %; p=0.02 ). Additionally, ultrasound-guided aspiration was more specific than fluoroscopy in diagnosing PJI using 2021 EBJIS criteria (100 % vs. 96 %; p=0.001 ).

Other important considerations in comparing fluoroscopic- and ultrasound-guided hip aspiration are cost and feasibility. Randelli et al. (2018) found that fluoroscopic-guided hip aspiration cost more than twice as much as ultrasound-guided hip aspiration. In their study, fluoroscopic-guided aspiration was performed in the operating room at an hourly cost of EUR 1000 with an average of 17 min required (EUR 283.33), whereas ultrasound was performed in the radiology department and cost comprised of investigation of the joint (EUR 36.55/patient) and hip aspiration (EUR 28.50/patient). In both fluoroscopic- and ultrasound-guided aspiration, the cost of microbiological culture was EUR 60.25 and increased to EUR 81.70 if cultures were positive and sensitivities were performed. Thus the average total for fluoroscopic-guided aspiration was EUR 343.58 and ultrasound-guided aspiration was EUR 125.30 (Randelli et al., 2018). At our institution, fluoroscopic-guided aspiration is performed in the radiology suite, whereas ultrasound-guided aspiration is performed in the clinic setting. Thus, fluoroscopic-guided aspiration requires provider and location availability, whereas ultrasound-guided aspiration only requires provider availability. However, fewer providers at our institution are trained to perform ultrasound-guided aspiration than fluoroscopic aspiration which may impact availability. Further studies are needed to compare time requirements, availability, and cost analysis between fluoroscopy and ultrasound.

Ultrasound is a safer method of image-guidance compared to fluoroscopy due to lack of radiation or contrast exposure. Additionally, ultrasound provides the ability to directly visualize soft tissue structures. This includes both intraarticular fluid collections and extraarticular fluid and structures including the greater trochanter bursa, iliopsoas tendon/bursa, gluteal tendons, and iliotibial band (Randelli et al., 2018). Ultrasound is a dynamic modality which allows for direct visualization of the needle tip passing through these soft tissue structures and entry into the hip capsule in real time. Despite these advantages, the use of ultrasound can be limited by the soft tissue envelope in the case of obesity. With deeper structures, anatomic landmarks may appear less distinct, making needle positioning more difficult (Chiodo et al., 2018). Chiodo et al. (2018) describes using a longer 3.5 in. spinal needle and lower frequency, curvilinear probe in obese patients to better target deeper structures. In our study, no difference was seen in BMI between the fluoroscopy and ultrasound cohorts (31.9 vs. 30.3, p=0.096 ), however, this was not a variable that we controlled for in analysis. Further studies are needed to better define the limitations of obesity on imaging when aspirating a total hip arthroplasty.

There were several limitations to our study. First, this was a retrospective review which has the inherent potential for selection bias due to non-randomization. The decision to perform fluoroscopic- or ultrasound-guided aspiration was primarily based on provider preference and clinical availability. Providers have variable levels of experience and expertise with each modality, which could have contributed to their decision making and success rates. Second, there is no perfect method to diagnose infection. In our analyses using culture results, there was potential for contaminants to influence the results. Additionally, surgeons sent fewer intraoperative cultures when aspiration cultures were negative (Table 10), which leads to a lower likelihood of diagnosing infection based on intraoperative cultures in this group. In our analyses using 2018 MSIS-ICM criteria and 2021 EBJIS criteria, there was potential for incomplete data points available for calculation of an individual's score. For example, D-dimer, synovial LE, synovial CRP, and intraoperative histology are not routinely collected at our institution. Third, we did not exclude patients with recent antibiotic use or spacer implants, which may affect culture growth and aspirate results. Future studies are needed to better define these contributing factors.

Our study showed that ultrasound-guided aspiration of a total hip arthroplasty was more commonly successful and resulted in fewer dry aspirations than fluoroscopy. Ultrasound-guided aspiration yielded a larger volume of fluid than fluoroscopy, even when excluding dry aspirations. Ultrasound was more sensitive than fluoroscopy in diagnosing PJI using culture results, 2018 MSIS-ICM criteria, and 2021 EBJIS criteria. Therefore, we believe that ultrasound guidance should be considered the preferred technique to identify PJI in THA.

## Data Availability

Data are not publicly available due to Institutional Review Board policies on human subjects.

## Code availability

The code is not publicly available due to Institutional Review Board policies.
